# Lymphotoxin Signaling Is Initiated by the Viral Polymerase in HCV-linked Tumorigenesis

**DOI:** 10.1371/journal.ppat.1003234

**Published:** 2013-03-21

**Authors:** Yannick Simonin, Serena Vegna, Leila Akkari, Damien Grégoire, Etienne Antoine, Jacques Piette, Nicolas Floc'h, Patrice Lassus, Guann-Yi Yu, Arielle R. Rosenberg, Michael Karin, David Durantel, Urszula Hibner

**Affiliations:** 1 CNRS, UMR 5535, Institut de Génétique Moléculaire de Montpellier, Montpellier, France; 2 Université de Montpellier 2, Place Eugène Bataillon, Université Montpellier 1, 5 Bd Henry IV, Montpellier, France; 3 National Infectious Diseases and Vaccinology, National Health Research Institutes, Zhunan, Miaoli, Taiwan; 4 Université Paris Descartes, EA4474 “Hepatitis C Virology”, Paris, France; 5 Laboratory of Gene Regulation and Signal Transduction, Departments of Pharmacology and Pathology, School of Medicine, University of California San Diego, La Jolla, California, United States of America; 6 INSERM, U1052, Cancer Research Center of Lyon (CRCL), University of Lyon, Lyon, France; University of North Carolina at Chapel Hill School of Medicine, United States of America

## Abstract

Exposure to hepatitis C virus (HCV) typically results in chronic infection that leads to progressive liver disease ranging from mild inflammation to severe fibrosis and cirrhosis as well as primary liver cancer. HCV triggers innate immune signaling within the infected hepatocyte, a first step in mounting of the adaptive response against HCV infection. Persistent inflammation is strongly associated with liver tumorigenesis. The goal of our work was to investigate the initiation of the inflammatory processes triggered by HCV viral proteins in their host cell and their possible link with HCV-related liver cancer. We report a dramatic upregulation of the lymphotoxin signaling pathway and more specifically of lymphotoxin-β in tumors of the FL-N/35 HCV-transgenic mice. Lymphotoxin expression is accompanied by activation of NF-κB, neosynthesis of chemokines and intra-tumoral recruitment of mononuclear cells. Spectacularly, IKKβ inactivation in FL-N/35 mice drastically reduces tumor incidence. Activation of lymphotoxin-β pathway can be reproduced in several cellular models, including the full length replicon and HCV-infected primary human hepatocytes. We have identified NS5B, the HCV RNA dependent RNA polymerase, as the viral protein responsible for this phenotype and shown that pharmacological inhibition of its activity alleviates activation of the pro-inflammatory pathway. These results open new perspectives in understanding the inflammatory mechanisms linked to HCV infection and tumorigenesis.

## Author Summary

Hepatitis C affects nearly 200 million people worldwide. It results from the failure of the immune system to control the hepatitis C virus (HCV) replication and spread, leading to progressive liver disease that can culminate in fibrosis, cirrhosis and cancer. The inflammatory cells that infiltrate the diseased liver functionally contribute to fibrotic disease and cancer development by the release of potent soluble mediators that regulate cell survival and proliferation, angiogenesis, tissue remodelling, metabolism and genomic integrity. The goal of our work was to study the mechanisms of the initiation of the inflammatory process linked to HCV infection. We have shown that the presence of a single viral protein, namely NS5B, the RNA dependent RNA polymerase, promotes pro-inflammatory signaling. Moreover, inhibition of this pathway in HCV transgenic mice fully protects the animals from HCV-linked liver cancer. Our study contributes to a better understanding of the inflammatory mechanisms linked to HCV infection and thereby to tumorigenesis.

## Introduction

Persistent HCV infection affects about 170 million people worldwide [1] and is one of the most common causes of chronic liver disease [2]. Infected individuals typically suffer from chronic liver inflammation that can last several decades and lead to progressive fibrotic liver that can culminate in hepatic cirrhosis and hepatocellular carcinoma (HCC) (for review see [3]).

Inflammation is the first step of the immune response against HCV infection and as such is beneficial to the host. However, in most cases, the infection is not resolved, fuelling the long-term persistent inflammation, with its many deleterious effects (for review see [4]), including the onset and progression of cancer. Inflammatory cytokines and chemokines are key molecular players in these processes, both by direct signaling, by recruiting further immune cells and by orchestrating production of reactive oxygen species, with their associated risk of inducing DNA mutations (for review see [5], [6].

Although the molecular mechanisms underlying HCV-associated liver cancer remain poorly understood (for review see [7]), there is no doubt that persistent liver inflammation increases the risk of HCC development by providing diverse mediators that perturb tissue homeostasis, including reactive oxygen species [8] and aberrant expression of cytotoxic cytokines [9], [10], [11]. Interestingly, it has been reported that several HCV proteins, namely core, NS3 and NS5A, can induce expression of pro-inflammatory cytokines [12], [13], [14] through yet to be identified mechanisms.

Lymphotoxin-α (LTα) and lymphotoxin-β (LTβ), two members of the tumor necrosis factor (TNF) superfamily, are necessary for organogenesis and maintenance of lymphoid tissues [15], [16]. LTα is soluble whereas LTβ contains a transmembrane domain. In consequence, LT exist both as soluble homotrimers (LTα3) that engage TNF receptor (TNFR) 1 and TNFR2 and the herpes virus entry mediator receptor (HVEM) and as membrane-bound heterotrimers (LTα1β2 or LTα2β1) that activate LTβR [17], [18]. LTβR acts through activation of canonical and alternative NF-κB signaling to induce the expression of a subset of chemokines (for review see [19], [20]. It has been shown that HCV infection is associated with increased hepatic LT expression both *in vivo* and *in vitro* [10], [21] and that HCV core protein can interact with the cytoplasmic domain of LTβR, thus stimulating the NF-κB pathways [22], [23]. Moreover, HCV replication *in vitro* depends on components of the LTβR pathway [24] while an ectopic LT expression in transgenic mice gives rise to liver inflammation and HCC [21]. However, the molecular mechanisms responsible for switching on LT expression in the HCV-infected hepatocytes have not been elucidated.

Here we report that tumors of HCV transgenic mice (FL-N/35 lineage) exhibit constitutively active LTβR and NF-κB signaling. Inhibition of the canonical NF-κB pathway through hepatocyte-specific deletion of Ikkβ [25] fully protects the animals from HCV-linked HCC. We further show that the viral RNA polymerase, NS5B, either alone or in the context of the full complement of viral proteins, is sufficient to induce expression of LT and NF-κB -dependant expression of its downstream target, CXCL10. Our data identify NS5B, recently shown to induce cytokine expression in hepatocytes through an RNA-dependent mechanism [26], as an inducer of the LTβR pathway, and specifically of lymphotoxin beta expression. These findings suggest that inhibitors of lymphotoxin signaling together with viral RNA polymerase inhibitors can be used to reduce HCV induced liver inflammation and HCC risk

## Results

### Immune cell infiltration of FL-N/35 tumors

**Figure 1:**
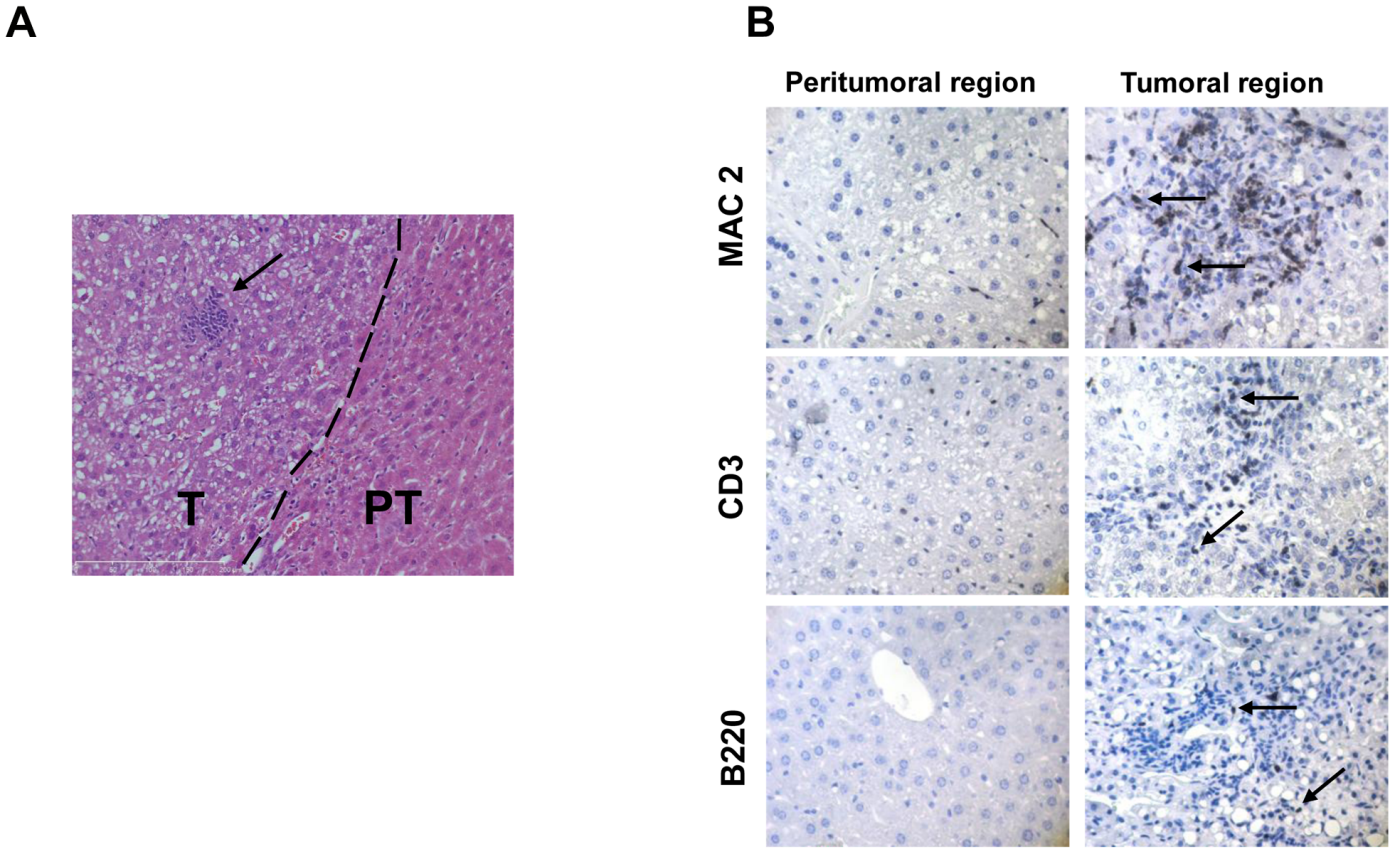
Immune cell infiltration in FL-N/35 tumors. Histological sections of FL-N/35 livers tumors. (A) Haematoxylin and eosin staining. Arrow indicates cellular infiltration. (B) Immunohistochemical staining of macrophages (MAC2), T lymphocyte (CD3) and B lymphocyte (B220). Arrows indicate positive cells. T: tumor, P: peritumoral.

FL-N/35 transgenic mice have a hepatocyte-targeted expression of the entire open reading frame (ORF) of the genotype 1b HCV, leading to expression of low levels of the full complement of viral proteins in the liver [27], [28]. In this model, HCV protein expression renders male mice at risk for liver tumorigenesis after one year of age [27]. Despite previous reports of lack of overt inflammation in the FL-N/35 animals, and because a vast majority of human HCV-linked HCC develops in necroinflammatory livers, we decided to reinvestigate a possible more subtle liver inflammatory phenotype of the FL-N/35 mice. In accordance with previously published observations [27], [29], prior to tumor development we detected only rare inflammatory foci, and no significant increase in either the number of inflammatory cells or proinflammatory cytokine expression in FL-N/35 livers compared to wild type mice (Figures S1 and S2). In contrast, multiple cellular infiltrations were present in FL-N/35 tumors (Figure 1A). The infiltrates were polymorphic and more specifically contained macrophages as well as B and T lymphocytes (Figure 1B).

### Lymphotoxin expression in FL-N/35 tumors

**Figure 2:**
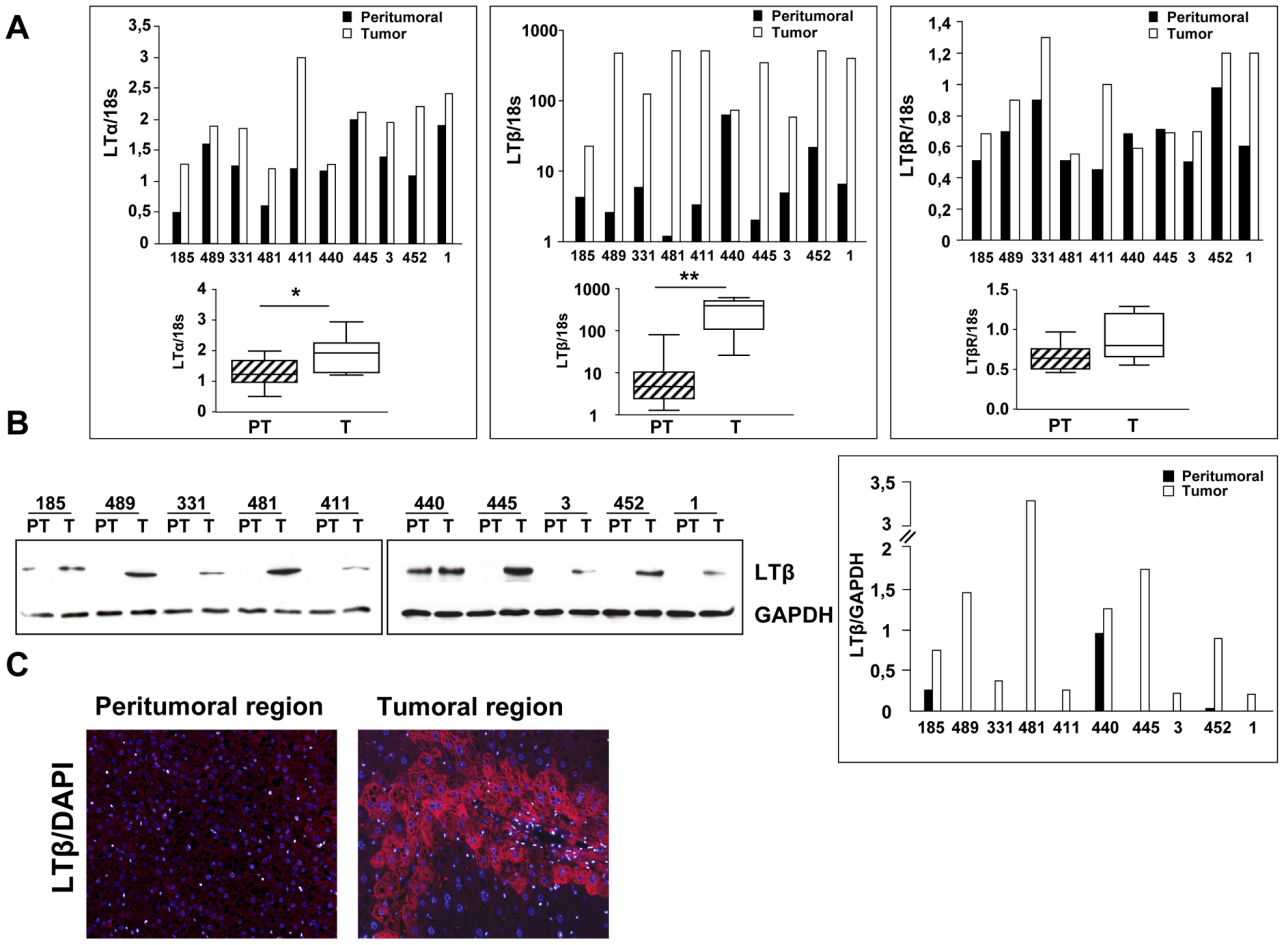
Lymphotoxin expression in FL-N/35 tumors. RNA and proteins extracted from FL-N/35 tumors and corresponding peritumoral areas were analyzed by RT-qPCR and by immunoblotting, respectively. (A) RT-qPCR analysis of LTα, LTβ and LTβR mRNA normalized to 18S ribosomal RNA. Numbers below bars identify the animals analyzed. Results were analyzed by Wilcoxon matched-pairs signed rank test (*p<0.05, **p<0.005). (B) Protein expression analyzed by immunoblotting with an anti-LTβ antibody (left panel) and corresponding quantification (right panel). Expression of a housekeeping gene, GAPDH, served as a loading control. Numbers correspond to animals analyzed. PT = peritumoral, T = tumoral tissue. (C) Immunofluorescence analysis of LTβ expression in peritumoral and tumoral areas of a typical tumor-bearing liver. DAPI staining was used to visualize nuclei.

**Figure 3:**
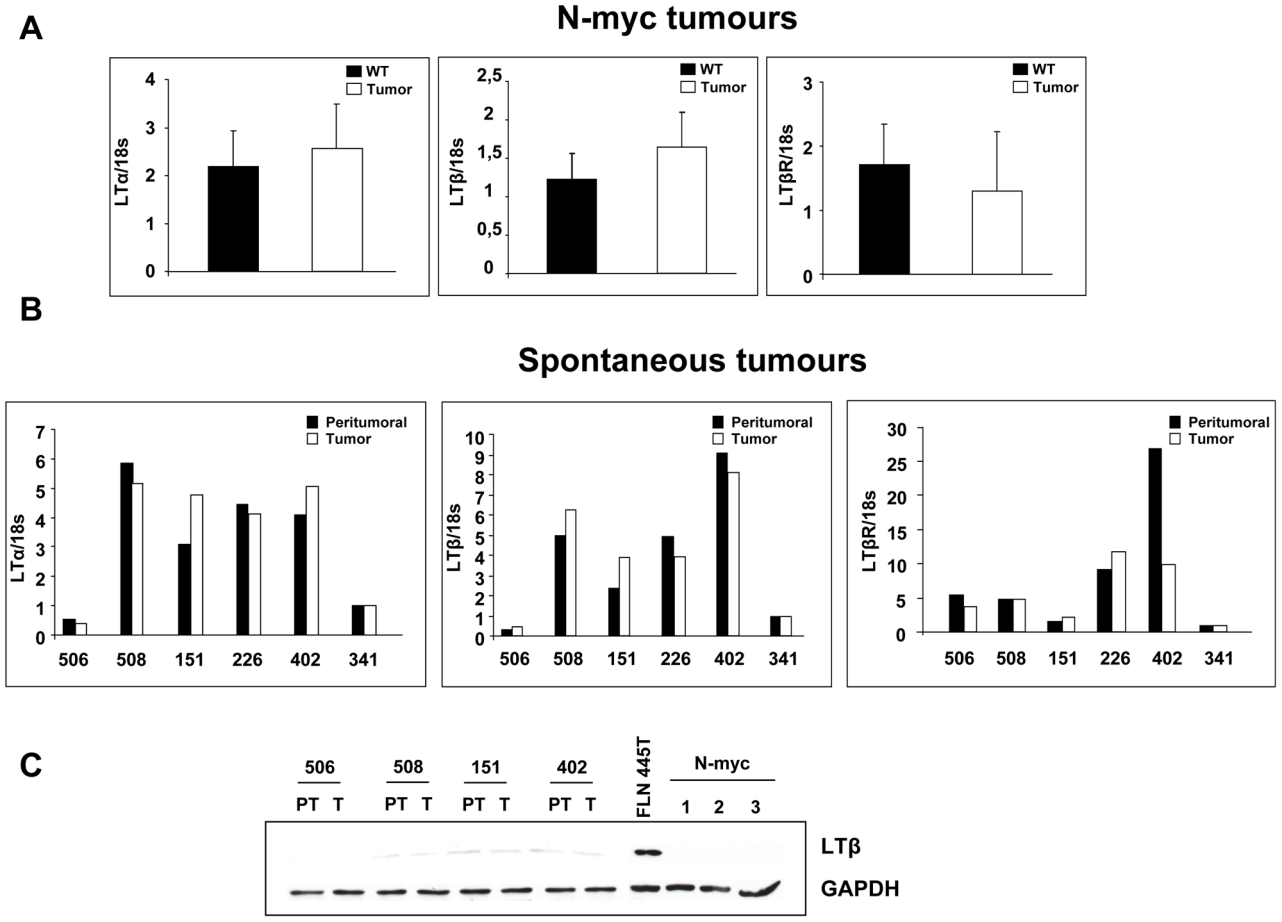
Lymphotoxins are not deregulated either in N-myc-driven or in spontaneous liver tumors. (A) RT-qPCR analysis of LTα, LTβ and LTβR mRNA expression in N-myc tumors and wild type mice from the same breeding. Quantification was performed on three different mice. (B) RT-qPCR analysis of LTα, LTβ and LTβR mRNA expression in spontaneous tumors. 18S RNA expression was used as a reference. (C) Western blot analysis of LTβ in spontaneous and N-myc tumors. FLN 445 tumor was used as positive control. Expression of a housekeeping gene, GAPDH, served as a loading control. Numbers identify the animals analyzed.

It has been reported that activation of inflammatory signaling triggered by LTβR gives rise to hepatocellular tumors in mice [21]. To investigate whether this pathway is instrumental in HCV-related tumorigenesis in FL-N/35 animals, we studied the expression of several of its key components. Quantitative RT-PCR analysis showed a dramatic increase in LTβ expression in all FL-N/35 tumors analyzed (n = 10). LTα expression was also increased in most tumors, albeit to a lesser extent, while LTβR levels did not differ significantly between tumoral and peritumoral samples (Figure 2A). Tumor-specific augmentation of LTβ expression was confirmed at the protein level (Figure 2B), while immunofluorescence staining showed that hepatocytes were the major source of this cytokine (Figure 2C). Strong LTβ expression was specific to HCV-linked liver tumors, as it was not increased in N-myc driven tumors of WHV/N-myc2 transgenic mice [30] (Figures 3A and 3C). Reinforcing this result, there was no increase in LT expression in rare spontaneous liver tumors arising in animals of the same genetic background as FL-N/35 mice (Figures 3B and 3C). In addition to LTβ, several pro-inflammatory cytokines, notably TNFα, IL6 and Il1β (Figure S3) were mildly, but significantly increased in HCV-related tumors, while changes of interferons α and β expression (Figure S4) did not reach statistical significance. Altogether, these results suggest a specific link between LTβ and HCV-related tumorigenesis.

Increased LT expression has been reported in many human hepatic pathologies, including HCC of different etiologies [10], [21]. We have confirmed these observations by showing significant increase of LTβ in tumoral and peri-tumoral samples of patients carrying HCC of either HCV or alcohol related cirrhosis (Figure S5A). Importantly, hepatocytes are a major source of this cytokine in the diseased liver (Figure S5B).

### NF-κB signaling is activated in FL-N/35 tumors

**Figure 4:**
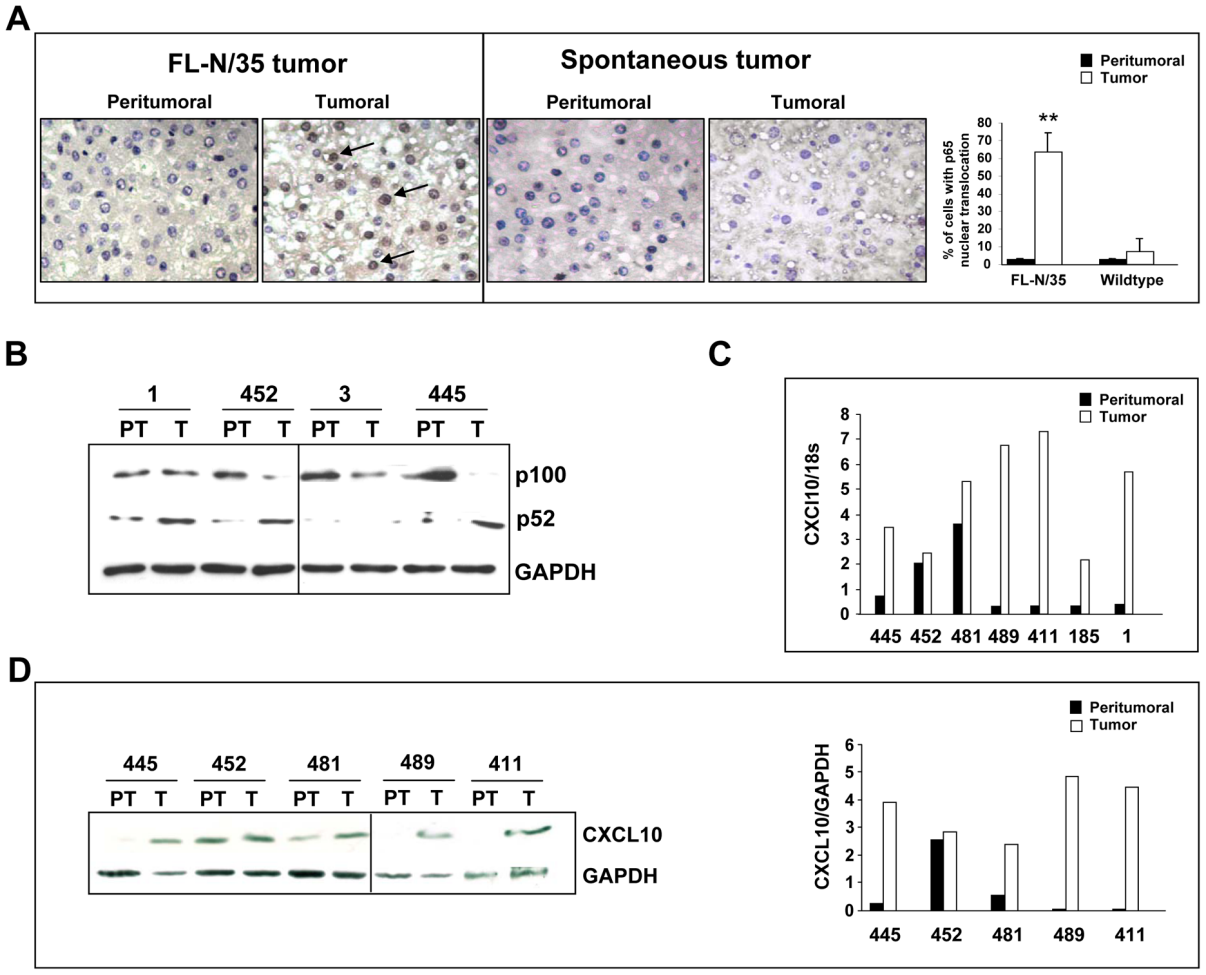
NF-κB activation in FL-N/35 tumors. (A) Immunohistochemical staining of p65 NF-κB subunit (brown) in FL-N/35 and spontaneous tumors. Arrows point to nuclear localization of p65, indicative of NF-κB activation. Nuclei are counterstained in blue. Quantification of p65 translocation is presented as mean+/− SEM of three independent experiments (**p<0.001). (B) p100, p52 protein levels in tumors and peritumoral regions of FL-N/35 mice livers. Processing of p100 to p52 is indicative of noncanonical NF-κB signaling. (C). RT-qPCR analysis of CXCL10 mRNA expression in FL-N/35 tumors and corresponding peritumoral areas. (D) CXCL10 protein levels and corresponding quantification in tumors and peritumoral regions of FL-N/35 mice. PT = peritumoral, T: tumor. Numbers identify animals analyzed.

LTβR signals through canonical and alternative NF-κB pathways to induce expression of several pro-inflammatory chemokines that act to recruit immune cells (for review see [18], [20]). To determine if LTβ upregulation is associated with activation of NF-κB signaling in the FL-N/35 tumors, we first investigated RelA (p65) localization in livers of tumor-bearing animals. Nuclear translocation of p65, indicative of canonical NF-κB activation, was detected in over 60% of tumoral hepatocytes, while less than 5% of peritumoral cells were positive in this assay, suggesting that NF-κB signaling was indeed activated in cells expressing LTβ (Figure 4A). In contrast, NF-κB was not activated in spontaneous liver tumors (Figure 4A). Next we assayed for activation of the alternative NF-κB signaling by visualizing cleavage of p100 into the mature p52 form of NF-κB. In agreement with previous reports of LT mode of action [19], the alternative NF-κB signaling was also activated in the HCV-related mouse tumors (Figure 4B). Moreover, the majority of tested tumors showed a strong increase of expression of CXCL10 (Figure 4C and 4D), an inflammatory chemokine downstream of LTβR (for review see [31]; [32]). Altogether these data suggest that increased LTβ expression in HCV-linked tumors leads to activation LTβR pathway of proinflammatory signaling.

### IKKβ-dependent NF-κB signaling is required for FL-N/35 tumorigenesis

**Figure 5:**
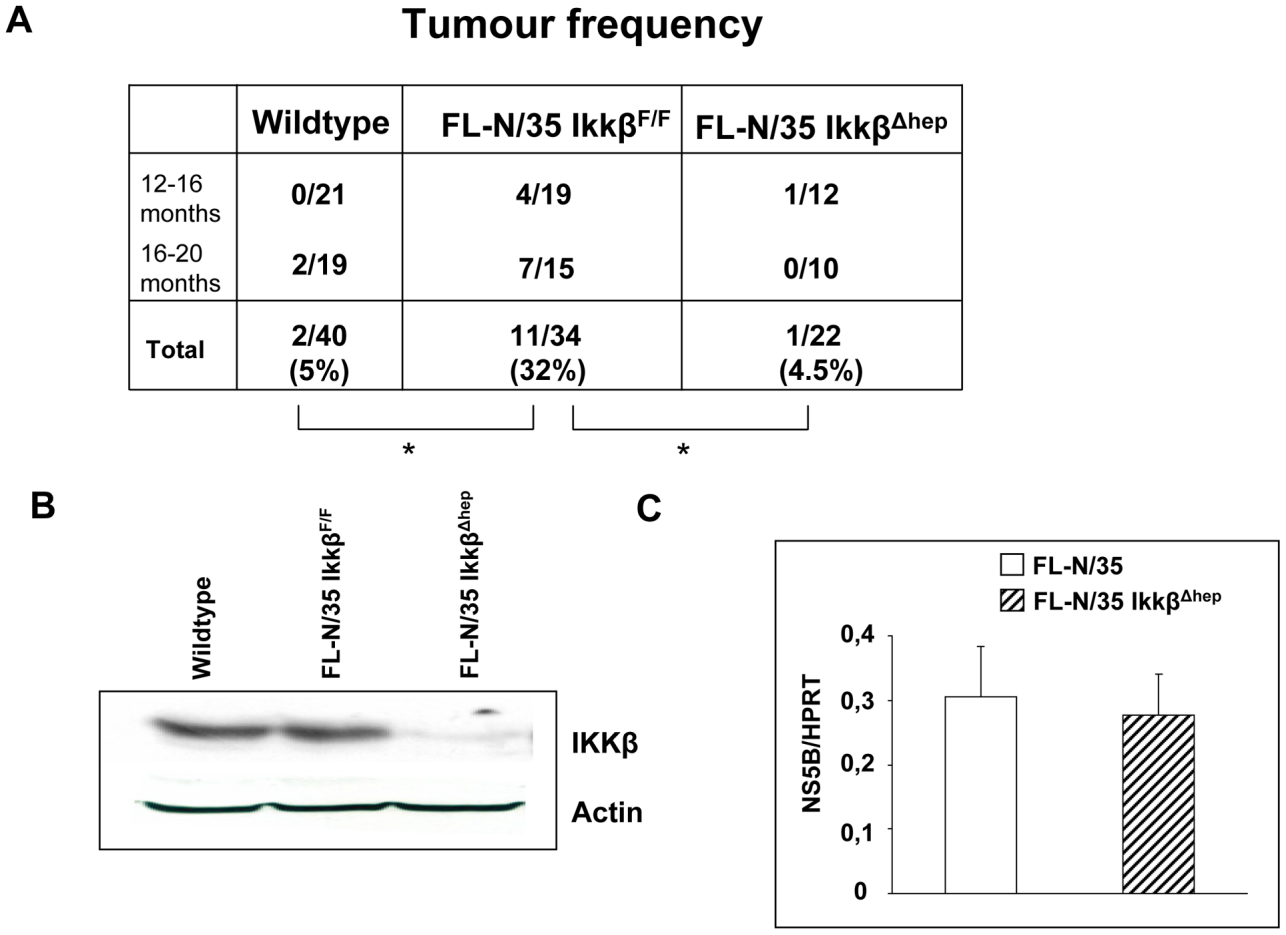
Invalidation of the canonical NF-κB signaling reduces tumor incidence in HCV transgenic mice. (A) Tumor incidence in control, FL-N/35 Ikkβ^Flox/Flox^ and FL-N/35 Ikkβ^Δhep^ male mice as a function of age. All animals are of the same mixed C57Bl/6/C3H genetic background. *p<0.02 (two sided Fisher's exact test). (B) IKKβ protein expression in wild type, FL-N/35Ikkβ^F/F^ and FL-N/35 Ikkβ^F/F^: Alb-Cre (FL-N/35 Ikkβ^Δhep^) mice. (C) RT-qPCR analysis of NS5B mRNA expression in FL-N/35 and FL-N/35 Ikkβ**^Δhep^** mice. Student's test showed no significant differences between the two groups.

While the role of canonical and alternative NF-κB signaling in liver carcinogenesis is complex (for review see [11]; [25]; [33]), it was suggested that the canonical NF-κB pathway is instrumental in relaying the oncogenic signal provided by LTβR activation [21]. This signal depends on the IKKβ catalytic subunit of the IκB kinase complex [34]. To determine if this scenario is operational in HCV-linked tumors, we crossed FL-N/35 mice with hepatocyte-specific IKKβ-deficient animals (IKKβ^Δhep^) [35]. As previously reported [27], HCV transgenic mice carrying wild type Ikkβ alleles are tumor-prone, with 30% of males developing hepatocellular adenoma and carcinoma after 12 months of age (Figure 5A). In the genetic background compatible with HCV-related liver tumorigenesis ([28] and our unpublished data), we routinely observe spontaneous liver tumors in about 5% of over one year old males. Strikingly, in FL-N/35/IKKβ^Δhep^ mice, in which Ikkβ deletion was confirmed by western blot (Figure 5B) and which express similar levels of HCV RNA that the control FL-N/35 animals (Figure 5C), the frequency of tumor formation was indistinguishable from wt non-transgenic males (Figure 5A) and, similarly to spontaneous lesions, the single hepatic tumor that appeared in this cohort was negative for LTβ expression (not shown). Thus, invalidation of IKKβ-dependent canonical NF-κB signaling blocks HCV-related liver tumorigenesis in the FL-N/35 model.

### Molecular mechanism of LT induction by HCV proteins

**Figure 6:**
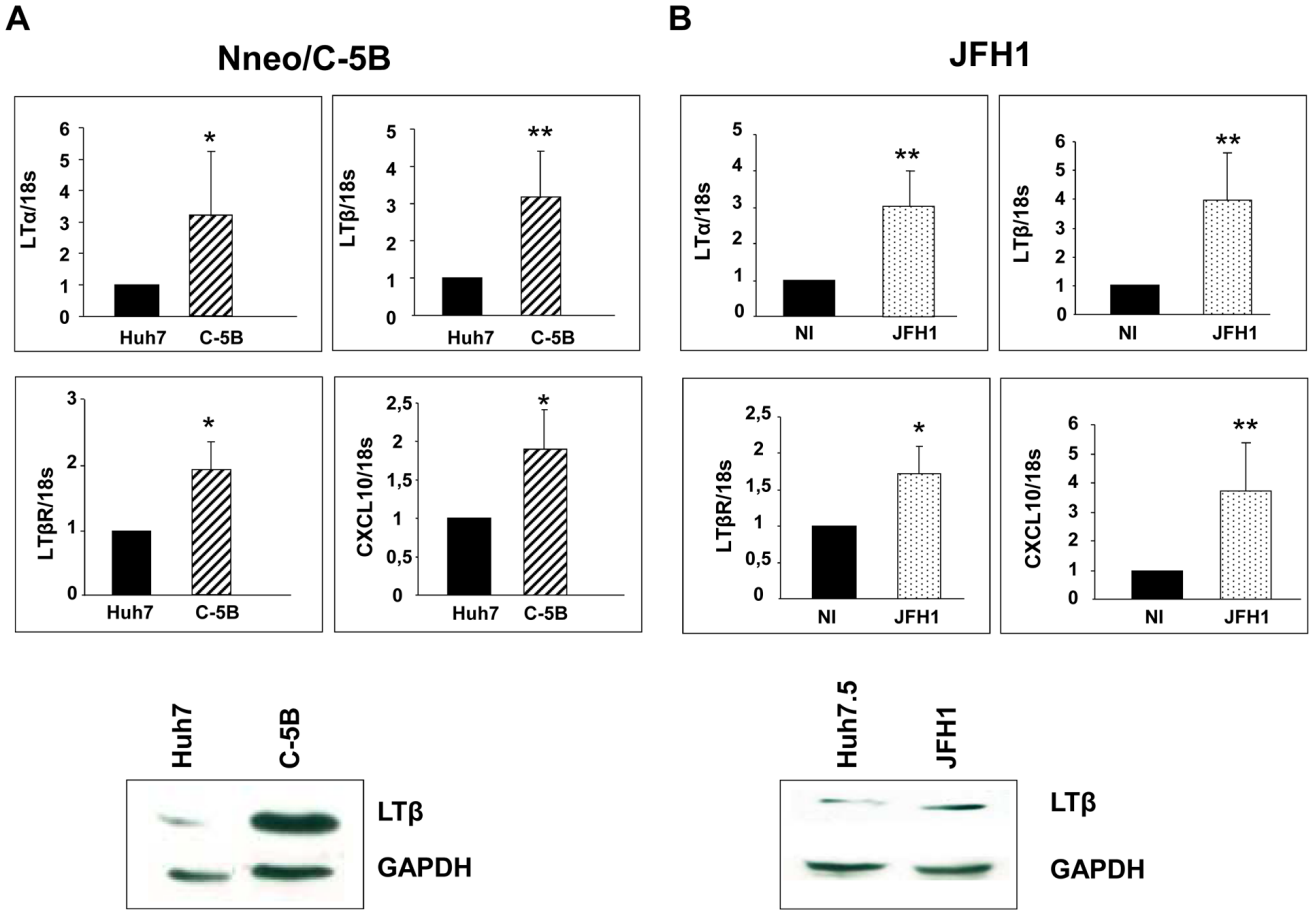
Upregulation of lymphotoxin signaling in HCV cellular models. Protein and RNA extracts from exponentially growing full-length (Nneo/C-5B) replicon lines, the infectious HCV model and the corresponding control cell lines, Huh7 and Huh7.5.1, respectively, were analyzed by RT-qPCR and by immunoblotting. (A) Analysis of LTα, LTβ, LTβR and CXCL10 mRNA expression and representative protein expression of LTβ in the Nneo/C-5B replicon propagated in Huh7 cells and in JFH1-infected Huh7.5.1 cells (B) Representative immunoblots of 3 independent experiments are shown. Where appropriate, results are presented as mean+/− SEM of 3 separate experiments (*p<0.01, **p<0.001).

To investigate the mechanism of LTβ induction by HCV proteins, we turned to a full-length HCV replicon propagated in Huh7 human hepatoma cells: the Nneo/C-5B model [36]. The replicon-containing cells expressed significantly more LTα, LTβ and, to a lesser extent, LTβR, compared to the parental Huh7 cells (Figure 6A). As in tumors from HCV transgenic mice, expression of CXCL10 was also induced in the Nneo/C-5B cells, suggesting that pro-inflammatory signaling cascade was activated. Moreover, productive infection of Huh-7.5.1 cells with JFH1-derived Con1/C3 HCV [37], [38] gave rise to a similar pattern of inflammatory signaling (Figure 6B).

**Figure 7:**
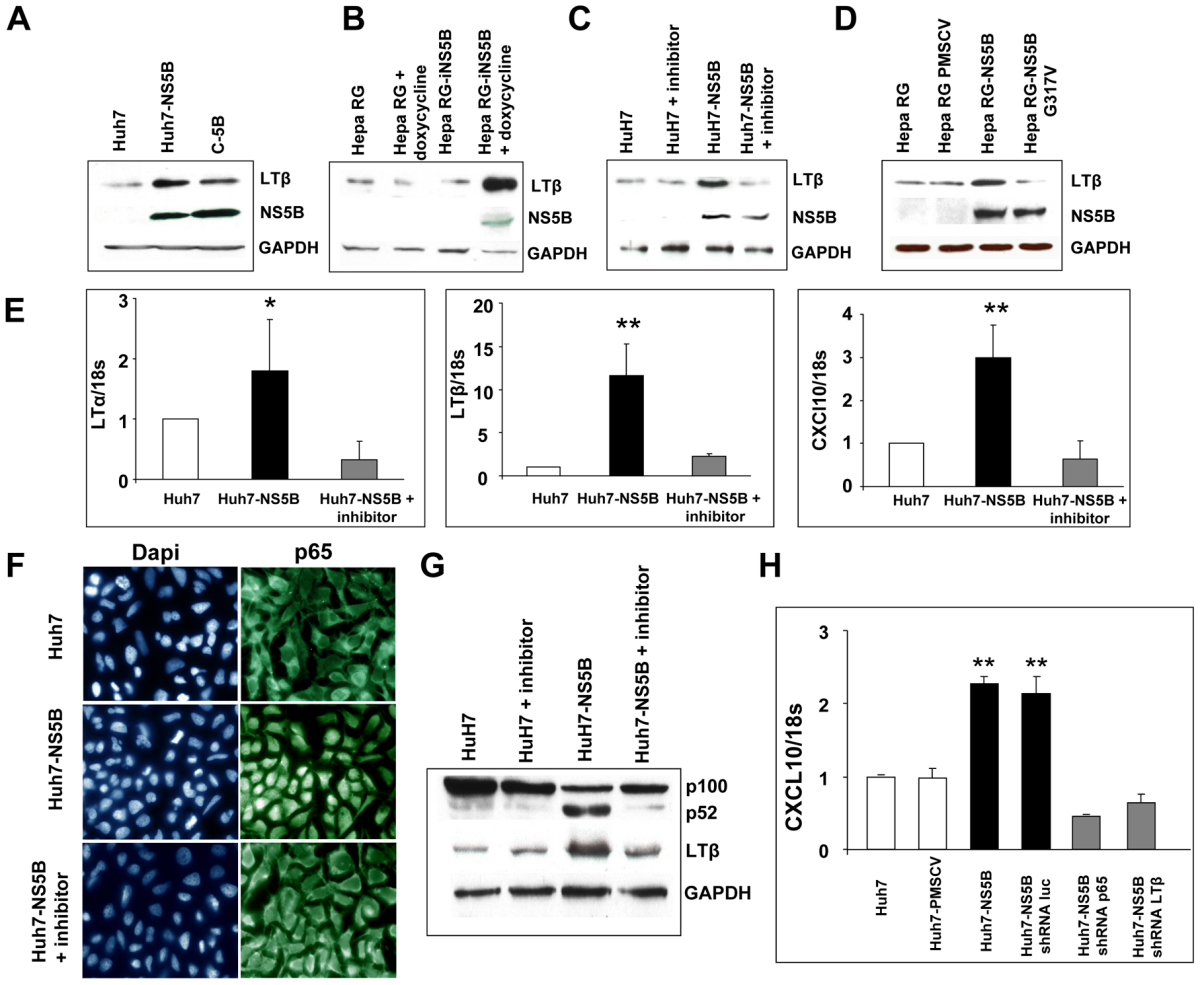
NS5B enzymatic activity is required for activation of lymphotoxin expression and signaling. (A) Expression of LTβ protein in exponentially growing Huh7 cells, Huh7 stably expressing NS5B (Huh7-NS5B) and Nneo/C-5B replicon (C-5B) (B) Expression of LTβ protein in HepaRG and HepaRG-NS5B doxycycline-inducible cells (HepaRG-iNS5B). (C) Expression of LTβ protein in exponentially growing Huh7 cells and in Huh7 stably expressing NS5B, treated with the NS5B polymerase inhibitor 2′-C-Methylcytidine, as indicated. (D) Expression of LTβ protein in HepaRG, HepaRG-PMSCV, HepaRG-NS5B and HepaRG-NS5B G317V. (E) RT-qPCR analysis of LTα, LTβ, LTβR and CXCL10 mRNA expression in Huh7 and Huh7-NS5B treated or not by 2′-C-Methylcytidine. 18S rRNA served as normalization standard. (F) Immunofluorescence analysis of p65 nuclear translocation in Huh7 cells and Huh7-NS5B treated or not with 2′-C-Methylcytidine. (G) Expression of p100, p52 and LTβ proteins in Huh7 cells and Huh7 stably expressing NS5B treated or not with 2′-C-Methylcytidine. (H) RT-qPCR analysis of CXCL10 mRNA expression. Parental and NS5B-expressing Huh7 cells were transduced with retroviral vectors encoding shRNA directed against p65, LTβ or Firefly luciferase as control. Where appropriate, results are presented as mean+/− SEM of three separate experiments (student test, *p<0.01, **p<0.001).

While the HCV proteins are organized in an endoplasmic reticulum-associated multiprotein complex [39], isolated viral proteins maintain some activities that may be relevant to the physiopathology of viral infection. To determine if LT pathway activation could be related to a specific viral protein, we established stable polyclonal Huh7 populations in which expression of individual HCV proteins was driven by a heterologous promoter. Out of the five proteins tested (core, NS3, NS4A, NS5A and NS5B), only NS5B, the viral RNA-dependent RNA polymerase, reproduced the increase of LTβ expression (Figure 7A, 7D, Figure S6). This result was not a peculiarity of the cellular model used, since it was confirmed in HepaRG-tetNS5B cells, which are human immature hepatocytes closely resembling primary cells [40] with doxycycline-regulated expression of NS5B (Figure 7B). Interestingly, in contrast to most models used in this study, which are based on HCV proteins of the 1b genotype, the infectious JFH-1-based model and the HepaRG-tetNS5B express the genotype 2a NS5B, demonstrating that the observed phenotype is not restricted to a single viral isolate.

Next we asked if the enzymatic activity of NS5B was required for LTβ upregulation. Huh7 cells constitutively expressing NS5B were treated with 2′-C-Methylcytidine, a pharmacological inhibitor of RNA-dependent RNA polymerase activity [41], [42], [43]. While this treatment had no effect on NS5B expression, it abrogated upregulation of LTβ, LTα and CXCL10 (Figure 7C and 7E). Similarly, expression of a catalytically inactive mutant, NS5B G317V, [44] in HepaRG cells did not activate LTβ synthesis (Figure 7D). Importantly, enzymatic activity of NS5B was also required for activation of both the canonical and the alternative NF-κB signaling (Figure 7 F and G).

Finally, we studied the functional relationship between NF-κB and LT signaling and their downstream effector, the CXCL10 chemokine. We used shRNAs to silence expression of either the p65 NF-κB subunit or LTβ in Huh7-NS5B cells. Silencing of either of these genes fully abrogated CXCL10 induction by NS5B (Figures 7H, Figure S7). Taken together, our results strongly support the notion that NS5B activity, in the absence of viral RNA, gives rise to increased lymphotoxin expression, which in turn activates a NF-κB-dependent pro-inflammatory signaling.

## Discussion

Persistent HCV infection is a major cause of chronic liver disease. In particular chronic inflammation, resulting from continuous immune response against infected hepatocytes, is associated with necro-inflammatory changes, liver fibrosis and cirrhosis and HCC development (for review see [45]). The molecular mechanisms involved in initiation and in fuelling of this process, sometimes over very long periods, are still incompletely understood (for review see [7]). In this report we show an upregulation of a pro-inflammatory cytokine, LTβ, and its downstream targets, NF-κB and CXCL10, in HCV-related tumors and in several cellular models based on expression of HCV proteins. The most spectacular alteration of this inflammatory signaling pathway was a very strong upregulation of LTβ expression in nine out of ten liver tumors of transgenic mice with liver-targeted expression of HCV proteins. The one exception (animal 440 in Fig. 2) had high levels of LTβ transcripts and protein both in the tumoral and peri-tumoral liver samples, suggestive of an ongoing inflammation unrelated to HCV. Augmented LTβ expression was also observed in several hepatocyte cell lines harboring the totality or a subset of HCV proteins or solely NS5B, the RNA dependent RNA polymerase. However, it was not detectable in non-tumoral regions of FL-N/35 transgenic livers despite the presence of detectable viral RNA transcripts. In this context it is noteworthy that while efficient cytokine induction by NS5B requires high levels of the enzyme [26], the expression of HCV proteins is typically over 10–100 fold higher in cellular models compared to the transgenic mouse livers analyzed here [46], probably accounting for lack of LT expression in the livers of the FL-N/35 animals. Interestingly, the level of viral RNA in mouse tumors is comparable to that found in peritumoral liver (data not shown). Although we cannot exclude possible variations of NS5B protein expression between the non-tumoral and the tumoral tissues, as well as within individual cells, our data suggest that LT activation might not initiate tumorigenesis, but rather contributes to tumor progression in this animal model. Indeed, strong LTβ expression in 100% of tumors together with complete abrogation of HCV-linked tumorigenesis in animals invalidated for canonical NF-κB signaling, which acts both as an upstream activator and a downstream effector of LT pathway, prompt us to speculate that an autoregulatory loop involving LT and NF-κB might exist in HCV-linked HCC.

A previous report described strong activation of several additional inflammatory cytokines in mouse livers with orthotopic expression of NS5B [26]. In our experimental set up we detected only a mild, albeit significant, expression of TNFα, Il6 and Il1β and no significant increase in type I interferon in the mouse tumors. This apparent discrepancy between the two studies is once again most likely due to very different levels of expression of NS5B, which in our experimental model is at least an order of magnitude lower and probably closer to the physiological levels present in the majority of chronic hepatitis C patients.

LT exists predominantly as a membrane bound heterotrimer of LTα and LTβ subunits with LTα1-β2 stoichiometry, which binds with high affinity to LTβR [17]. Importantly, increased expression of LTβ was previously described in patients, in the context of chronic hepatitis C-associated cirrhosis and HCC [10], [21], [47], supporting physiopathological relevance of our data.

LTβR activation gives rise to expression of several chemokines through canonical and alternative NF-κB signaling (for review see [19]. Interestingly, in the FL-N/35 HCV transgenic mouse model, where the tumors show strong activation of both LT and NF-κB, abrogation of the canonical NF-κB pathway by hepatocyte-specific IKKβ ablation, led to a dramatic decrease in tumor incidence, arguing for a major role of NF-κB in promoting tumorigenesis in the context of HCV. However, the role of NF-κB in liver carcinogenesis is complex, as it inhibits cell death-promoted tumorigenesis [25], [48], [49], while promoting inflammation-driven tumor-formation in Mdr2-deficient [33] and in LT-transgenic mice [21] and in xenografts of human HCC [50].

It is perhaps not surprising that NF-κB, with its many possible downstream effectors and activities [51] is endowed with both pro- and anti-tumorigenic activities that are dominant under different physiological contexts. However, it is noteworthy that our data, linking HCV with LT and NF-κB signaling in the context of hepatocellular tumorigenesis, are in full agreement with HCC development triggered by ectopic LT expression [21].

We have shown that increased LT expression in hepatocytes expressing viral proteins has functional consequences in that it leads to synthesis of CXCL10. This C-X-C chemokine is expressed by hepatocytes in chronic hepatitis C [21], [48], [52], [53], [54]. It is induced by LTβR via NF-κB [31], [55] and is considered as one of the main chemoattractors for tumor-infiltrating immune cells (for review see [56]). It is thus tempting to speculate that CXCL10, induced by HCV viral proteins via LTβR and NF-κB could initiate liver recruitment of hematopoietic cells as well as intratumoral cellular infiltrates.

Mechanistically, we have shown that NS5B, the viral RNA-dependent RNA polymerase, is sufficient to activate the LT pathway and therefore upregulate chemokine production. Although physiologically NS5B is part of a multiprotein replication complex, the isolated protein also has enzymatic activity [57]. Moreover, NS5B interacts with several cellular proteins, including transcriptional regulators such as Rb [58], [59], RNA cellular helicases such as p68, which modulates RNA structures and is involved in RNA splicing, processing, transcription and translation [60] and eIF4AII, an RNA-helicase translation initiation factor [61]. Furthermore, a recent study described the role of the RNA sequence encoding NS5B as a pathogen associated molecular pattern (PAMP) following RNase L cleavage [62]. While all these interactions might participate in triggering inflammatory signaling downstream of NS5B, our data indicating that the enzymatic activity of NS5B is essential for induction of LT expression suggest that the molecular mechanism of LTβR activation by HCV relies on RNA synthesis, most probably from cellular RNA templates [63]. Further biochemical experiments are needed to formally demonstrate this point.

These uncertainties notwithstanding, the discovery of LT pathway activation by NS5B and the fact that pharmacological inhibition of its enzymatic activity alleviates the pro-inflammatory phenotype, open new perspectives for understanding the inflammatory mechanisms linked to HCV infection. In particular these results suggest that LTβR signaling could be an interesting target for therapies aimed at curbing HCV-related liver inflammation, known to be a major risk factor for severe hepatic pathologies, including HCC.

## Materials and Methods

### Animals

FL-N/35 transgenic animals [27] and Ikkβ^F/F^:Alb-Cre (referred to as Ikkβ^Δhep^) [35] were bred and maintained according to the French institutional guidelines. Twelve to twenty month-old males were used in these experiments.

### Patient tissue samples

HCC and corresponding nontumoral tissues were obtained from resected specimens from patients treated at the University Hospitals of Bordeaux and Montpellier, France. Small pieces from tumoral and nontumoral livers were snap frozen in liquid nitrogen and stored at −80°C until use. In parallel, samples were fixed and processed for immunohistochemistry. Informed consent was obtained according to the institutional regulations.

### Cell culture and treatments

Huh7 cells were cultured in DMEM supplemented with 10% fetal bovine serum, 100 µg/ml streptomycin and 100 U/ml penicillin. 400 µg/ml of G418 were added to cells harboring the Nneo/C-5B replicons and 2 µg/ml of puromycin to Huh7-NS5B cells. HepaRG and HepaRG-NS5B tetracycline-inducible cells were grown in William's E medium supplemented with 10% fetal calf serum, 5 µg/ml insulin, 5.10^−5^ M hydrocortisone hemisuccinate, 100 units/ml penicillin, and 100 µg/ml streptomycin. When appropriate, cells were treated for 24 hours with 6 µg/ml of the NS5B inhibitors 2′-C-Methylcytidine from Santa Cruz Biotechnology (Heidelberg, Germany) or with 0.5 µg/ml of doxycycline from Sigma (St. Louis, MO).

### Generation of stably transfected cell lines

NS5B cDNA sequences from genotype 1b was subcloned in Myc-tagged pMSCV retroviral vectors as previously described [64]. ShRNA coding sequences were cloned in pSIREN-RetroQ (Clontech, Palo Alto, CA). Plasmids were transfected into 293T cells with jetPEI (Polyplus, Illkirch, France), according to the manufacturer's instructions. Supernatants were used to infect Huh 7 cells. Infection efficiencies of 80% were routinely obtained. Puromycin (2 µg/ml) and hygromycin (150 µg/ml) were used as selection agents.

The sense and antisense strands of shRNAs were :

LTβ : 5′- atccgcctctactgtctcgtcggctattcaagagatagccgacgagacagtagaggcttttttctcgagg -3′

3′- gcggagatgacagagcagccgataagttctctatcggctgctctgtcatctccgaaaaaagagctccttaa -5′

P65 (RelA) : 5′- gatccggccttaatagtagggtaagttttcaagagaaacttaccctactattaaggccttttttctcgag -3′

3′- gccggaattatcatcccattcaaaagttctctttgaatgggatgataattccggaaaaaagagctccttaa –5′

ShLuc, the shRNA directed against luciferase, comes from RNAi-Ready pSIRENRetroQ Retroviral Vector kit (Clontech)

### Generation of NS5B catalytic mutant

The point mutation G317V [45]. was introduced in the GDD motif of the NS5B gene by site-directed mutagenesis (QuikChange II XL, Agilent Technologies), using the following primers :

5′-GCTCGTGAACGTAGACGACCTTGTC-3′, 5′-GACAAGGTCGTCTACGTTCACGAGC-3′.

The specificity of the mutagenesis was verified by DNA sequencing of the entire coding sequence.

### Immunobloting

Western blots were performed as described previously [65]. Band intensities were quantified with the Gene Tools software (SynGene). Polyclonal rabbit antibodies anti-LTβ (ab 64835) and anti-NS5B (ab 35586) were from Abcam (Cambridge, UK). Polyclonal rabbit antibodies anti-p100/p52 (4882p) was from Ozyme (Saint-Quentin, France). Mouse monoclonal antibodies anti-IKKβ (clone 10AG2, Upstate) and anti-CXCL10 were respectively from Millipore (Temecula, CA, USA) and BD Biosciences (Oxford, UK).

### RNA isolation and analysis

Total RNA was isolated using an RNeasy Mini Kit (Qiagen, Germantown, MD, USA) including DNase treatment to remove possible genomic DNA contamination and used for first strand cDNA synthesis with random hexamers. Analyses were performed as described previously [65].

### Histology

Mice were sacrificed with an overdose of pentobarbital (Narconen, Basel, Switzerland) and perfused transcardially with 4% paraformaldehyde in phosphate-buffered saline (PBS). The liver was removed, post-fixed and embedded in Tissue-Tek OCT Compound. Sections of 4 µm were stained with haematoxylin and eosin and then mounted in Eukitt.

### Immunohistochemistry

Four micrometer sections were mounted on glass slides and stained using ABC Vectastain system from Vector laboratory (Burlingame, CA, USA). Monoclonal primary mouse antibodies for mice samples were anti-Mac 2, anti CD3 from eBioscience (San Diego, CA, USA) anti B220 from BD Biosciences (Oxford, UK) and p65 from Santa Cruz Biotechnology (Heidelberg, Germany). For human samples polyclonal rabbit antibodies anti-LTβ (ab 64835) was from Abcam (Cambridge, UK). Biotinylated secondary antibody was from Vector Laboratory (Burlingame, CA, USA). Control experiments were done in the absence of the primary antibody and were negative in all cases.

### Immunofluorescence

Cells were fixed in 4% paraformaldehyde, permeabilized in 0.1% Triton X-100, rinsed in phosphate-buffered saline, blocked with 1 mg/ml BSA and incubated with rabbit polyclonal anti-LTβ antibody from Abcam (Cambridge, UK) or with anti-p52 from Santa Cruz Biotechnology (Heidelberg, Germany) for 2 hours followed by anti-rabbit Alexa Fluor 488 for 1 hour. Samples were mounted with Fluorosave (Calbiochem, La Jolla, CA, USA) and analysed with a Zeiss fluorescent microscope equipped with a digital camera (Axiocam, Carl Zeiss, Oberkochen, Germany).

### Statistical analysis

Experiments were performed at least three times. Data are presented either from a representative experiment or as mean ± SEM. Comparisons between groups were analyzed by Student's t test or Wilcoxon matched-pairs signed rank test as indicated.

## Supporting Information

Figure S1 **FACS analysis of intrahepatic myeloid, B, T, NK and NKT cells from wild type and FL-N/35 mice.** Analyses were performed on FACS Canto II (BD Bioscience, Oxford, UK) using following antibodies: CDK4-FITC; NK1.1-PE; CD19-PrCP; CD3-PC7; CD11b-APC; CD8-AAF750. Student's test showed no significant differences for any of the cells assayed.
(TIF)

Figure S2 **Cytokine expression profiles in livers of FL-N/35 and wild type mice.** RNA extracted from livers bearing no tumours in seven transgenic and seven wt mice was analyzed by RT-qPCR for LTα, LTβ, LTβR, TNFα, IL6, IL1b, IL18 (A) and CCL2, CXCL10, CXCL1, CCL5 (B) mRNA and normalized to 18S rRNA. Student's test showed no significant differences for any of the assayed cytokines.
(TIF)

Figure S3 **Expression profiles of pro-inflammatory cytokines in FL-N/35 tumors.** RNA extracted from FL-N/35 tumors and corresponding peritumoral areas were analyzed by RT-qPCR for IL6, IL18, TNFα, IL1β and normalized to 18S rRNA. Numbers correspond to different animals studied. Results were analyzed by Wilcoxon matched-pairs signed rank test. (*p<0.05).
(TIF)

Figure S4 **Expression profiles of IFNα and IFNβ in FL-N/35 tumors.** RNA extracted from FL-N/35 tumors and corresponding peritumoral areas were analyzed by RT-qPCR for IFNα (A) and IFNβ (B) and normalized to HPRT mRNA. Numbers correspond to different animals studied. Results were analyzed by Wilcoxon matched-pairs signed rank test and showed no significant difference.
(TIF)

Figure S5 **LTβ expression in human hepatocellular carcinoma.** (A) RNA was extracted from frozen specimens of human tumours and the corresponding non-tumoral liver tissues. The level of LTβ was assessed by quantitative RT-PCR and normalized to 18S mRNA. (B) Immunohistochemical staining of LTβ (brown) in a healthy control liver (left panel) and in a peritumoral (middle panel) region and HCC (right panel) from the same HCV+ patient. PT = peritumoral, T = tumoral.
(TIF)

Figure S6 **LTβ expression in cell lines stably expressing individual HCV proteins.** Huh7 cells were transduced with retroviral vectors coding for myc-tagged HCV1b proteins NS3, NS4A, core and NS5A, as indicated. Viral proteins expression was revealed by immunoblotting with an anti-myc monoclonal antibody.
(TIF)

Figure S7 **p65 and LTβ are efficiently silenced by their cognate shRNA.** NS5B-expressing and parental Huh7 cells were transduced with retroviral vectors encoding shRNA for p65 (A) or LTβ (B) and protein expression was assayed by immunoblotting. GAPDH served as a loading control.
(TIF)
