# Supplementary figures and images for: Lymphotoxin Signaling Is Initiated by the Viral Polymerase in HCV-linked Tumorigenesis
Source: PLoS Pathog. 2013 Mar 21;9(3):e1003234. doi: 10.1371/journal.ppat.1003234 (PMC3605200; doi:10.1371/journal.ppat.1003234)

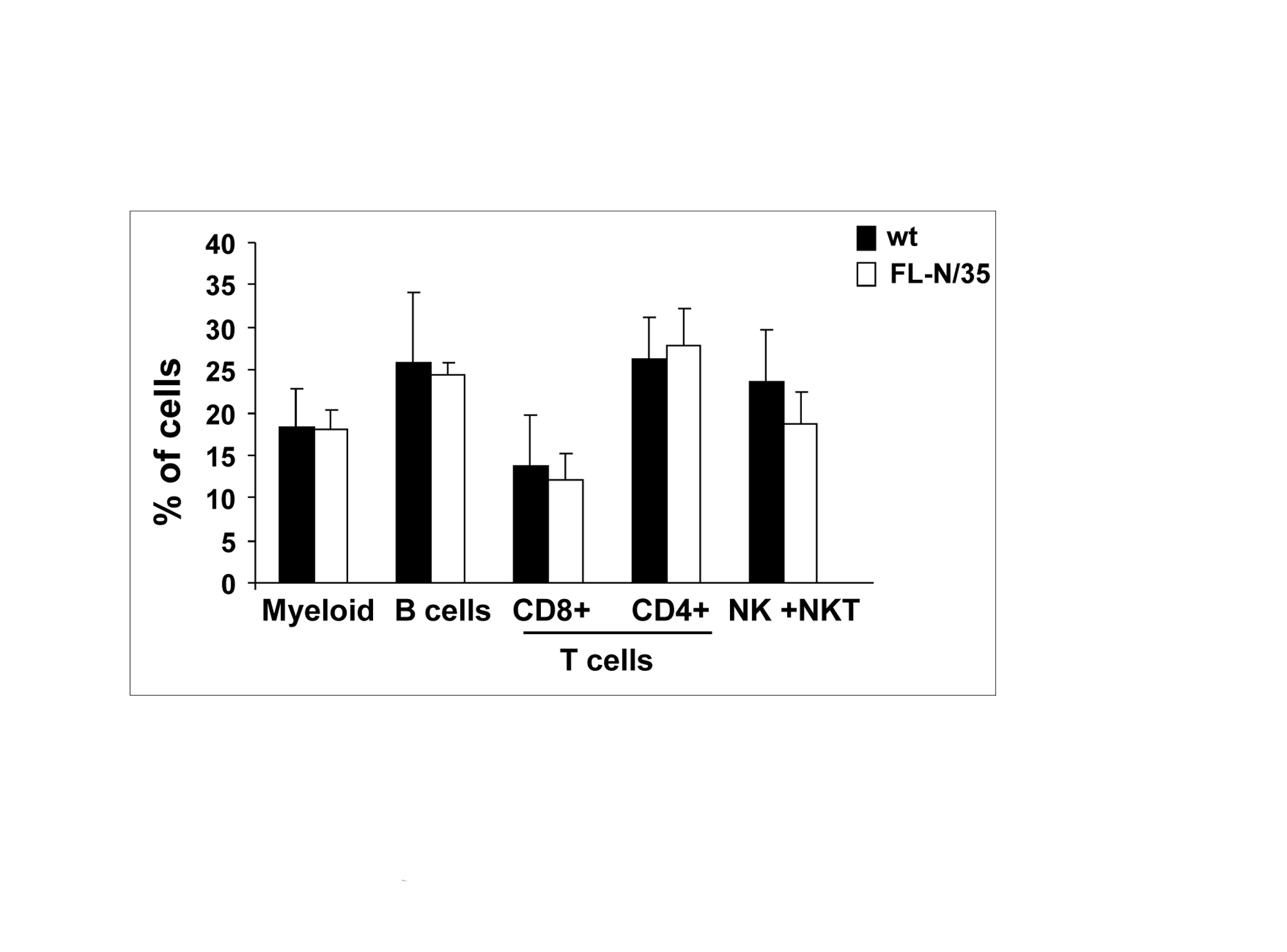

Supplement: Figure S1 — FACS analysis of intrahepatic myeloid, B, T, NK and NKT cells from wild type and FL-N/35 mice. Analyses were performed on FACS Canto II (BD Bioscience, Oxford, UK) using following antibodies: CDK4-FITC; NK1.1-PE; CD19-PrCP; CD3-PC7; CD11b-APC; CD8-AAF750. Student's test showed no significant differences for any of the cells assayed. (TIF) [file ppat.1003234.s001.tif]

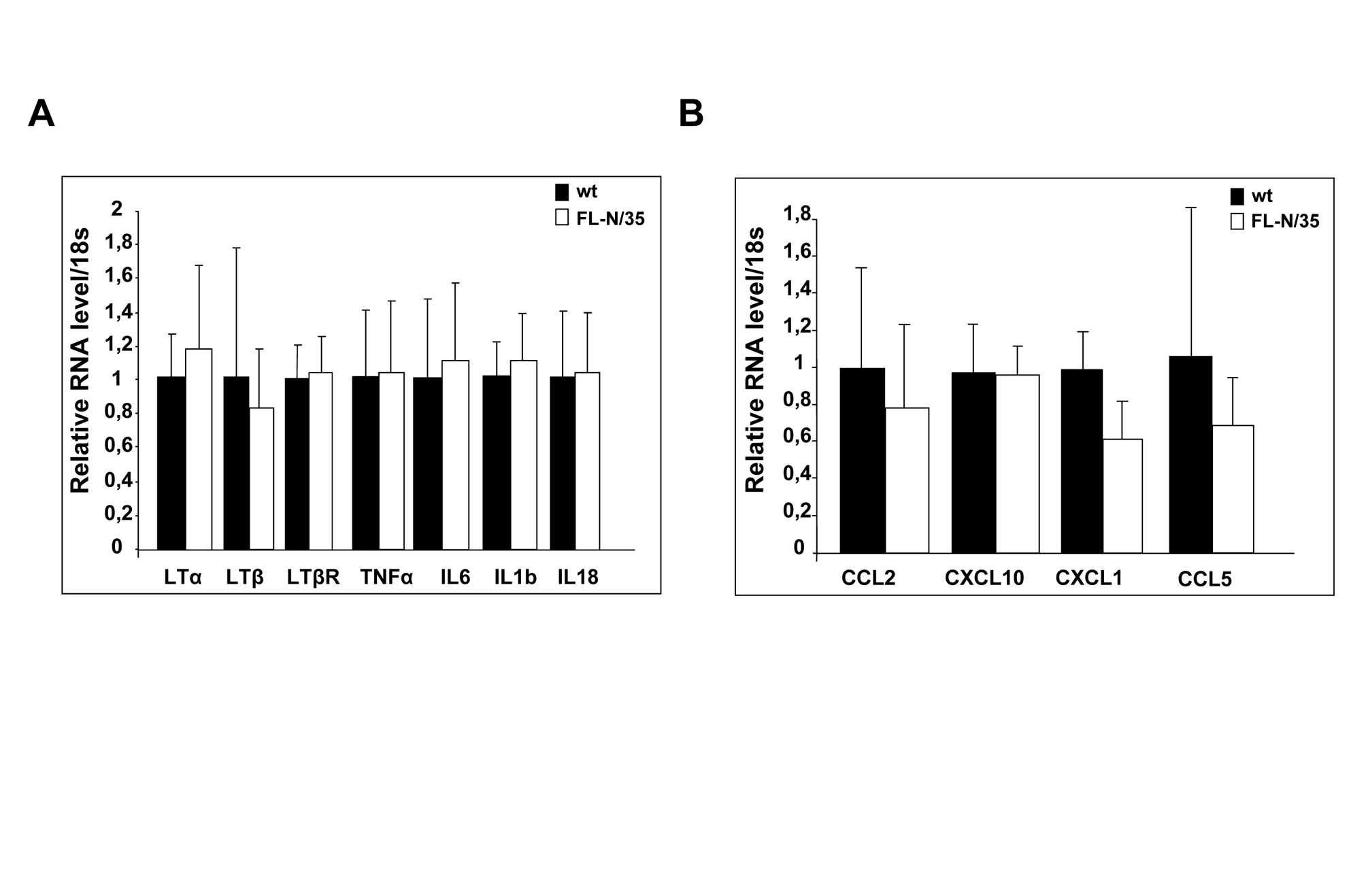

Supplement: Figure S2 — Cytokine expression profiles in livers of FL-N/35 and wild type mice. RNA extracted from livers bearing no tumours in seven transgenic and seven wt mice was analyzed by RT-qPCR for LTα, LTβ, LTβR, TNFα, IL6, IL1b, IL18 (A) and CCL2, CXCL10, CXCL1, CCL5 (B) mRNA and normalized to 18S rRNA. Student's test showed no significant differences for any of the assayed cytokines. (TIF) [file ppat.1003234.s002.tif]

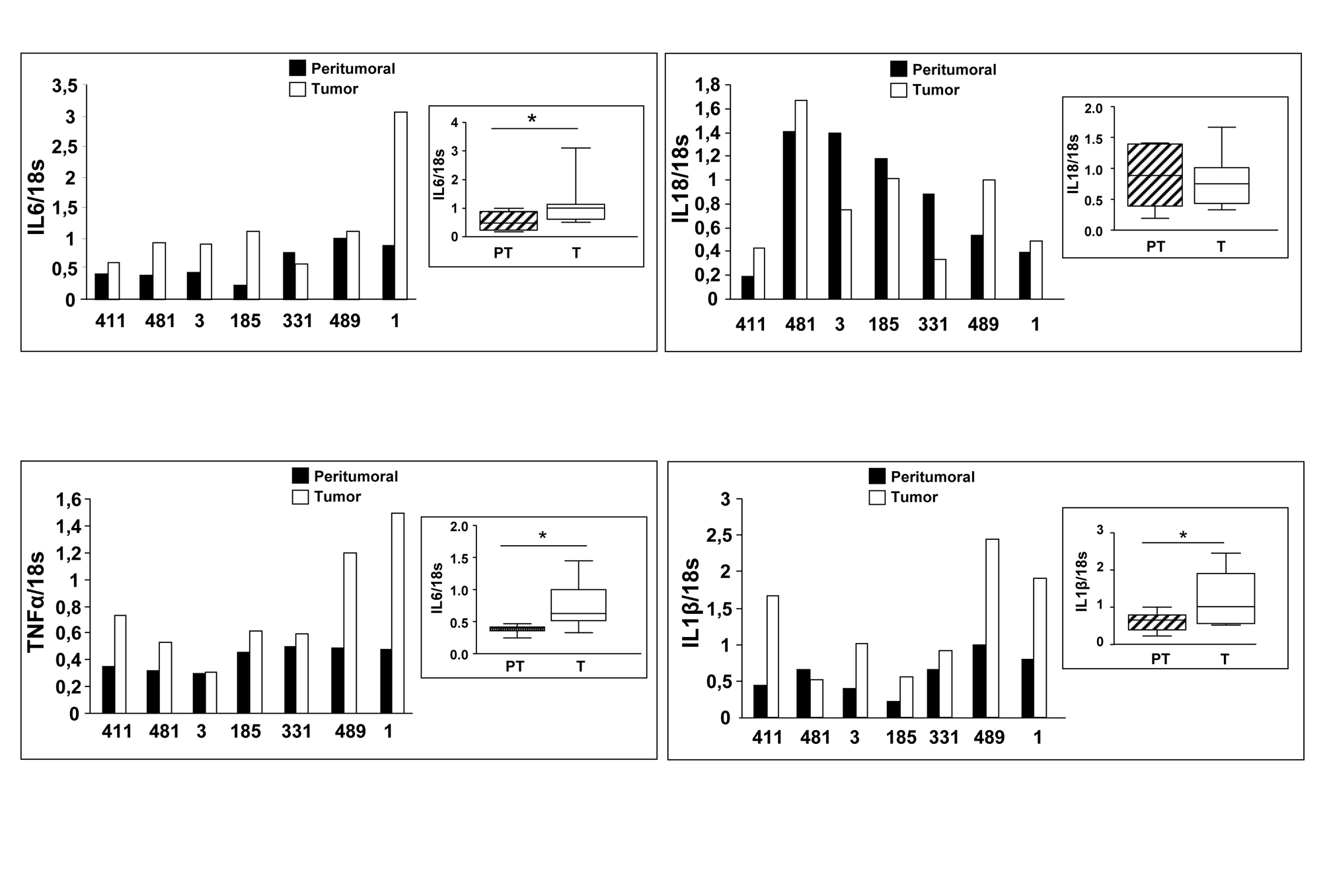

Supplement: Figure S3 — Expression profiles of pro-inflammatory cytokines in FL-N/35 tumors. RNA extracted from FL-N/35 tumors and corresponding peritumoral areas were analyzed by RT-qPCR for IL6, IL18, TNFα, IL1β and normalized to 18S rRNA. Numbers correspond to different animals studied. Results were analyzed by Wilcoxon matched-pairs signed rank test. (*p<0.05). (TIF) [file ppat.1003234.s003.tif]

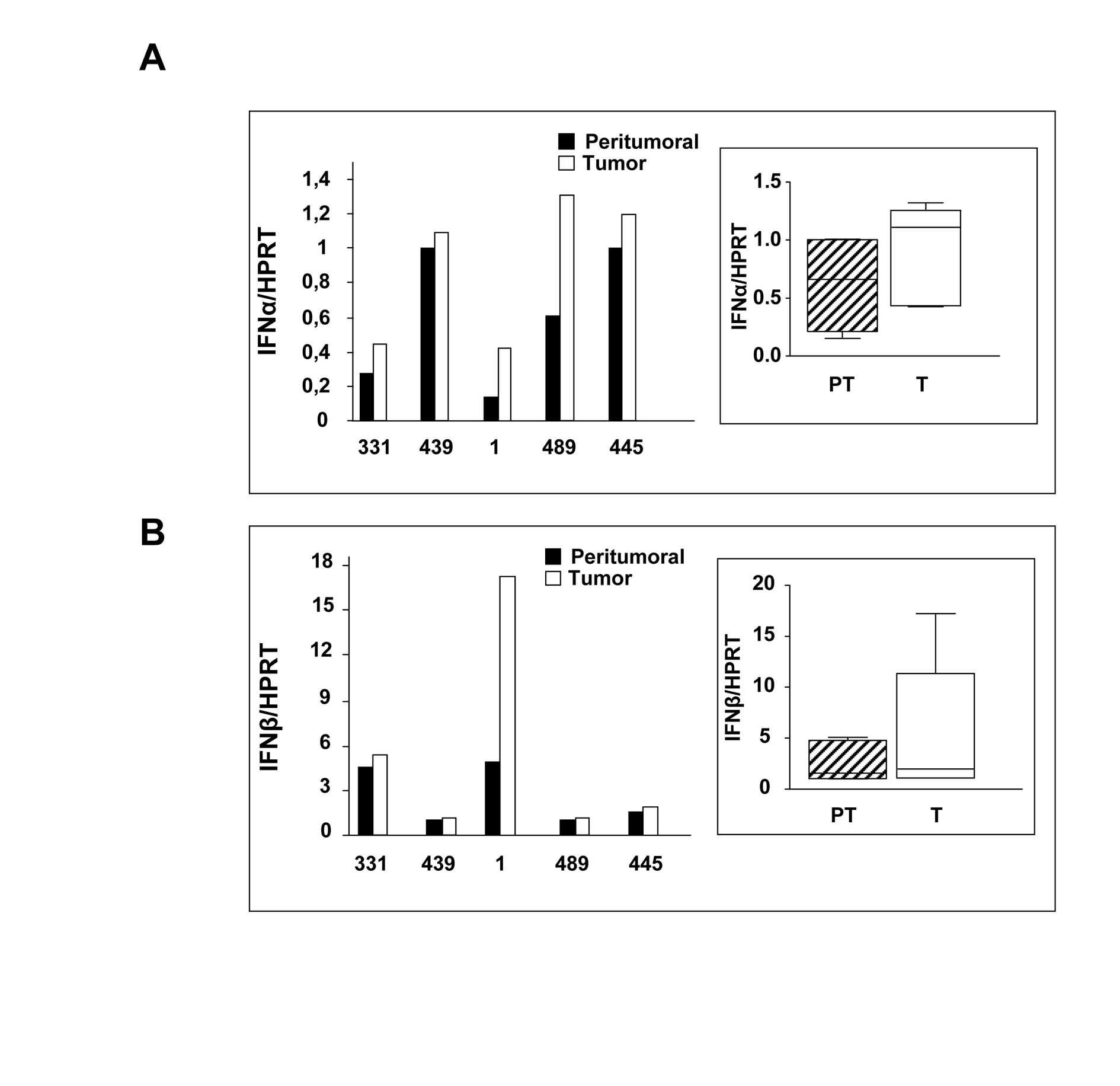

Supplement: Figure S4 — Expression profiles of IFNα and IFNβ in FL-N/35 tumors. RNA extracted from FL-N/35 tumors and corresponding peritumoral areas were analyzed by RT-qPCR for IFNα (A) and IFNβ (B) and normalized to HPRT mRNA. Numbers correspond to different animals studied. Results were analyzed by Wilcoxon matched-pairs signed rank test and showed no significant difference. (TIF) [file ppat.1003234.s004.tif]

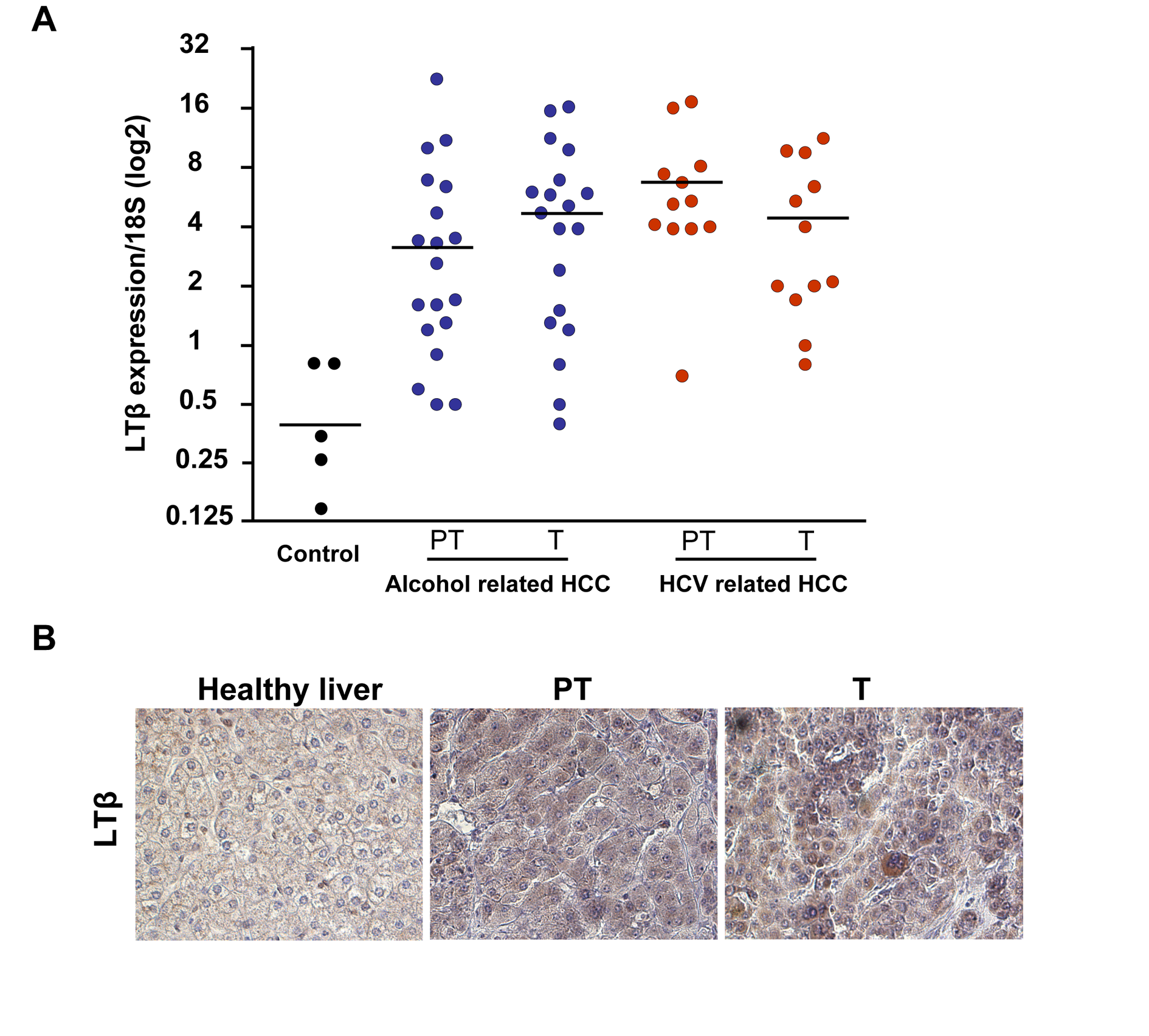

Supplement: Figure S5 — LTβ expression in human hepatocellular carcinoma. (A) RNA was extracted from frozen specimens of human tumours and the corresponding non-tumoral liver tissues. The level of LTβ was assessed by quantitative RT-PCR and normalized to 18S mRNA. (B) Immunohistochemical staining of LTβ (brown) in a healthy control liver (left panel) and in a peritumoral (middle panel) region and HCC (right panel) from the same HCV+ patient. PT = peritumoral, T = tumoral. (TIF) [file ppat.1003234.s005.tif]

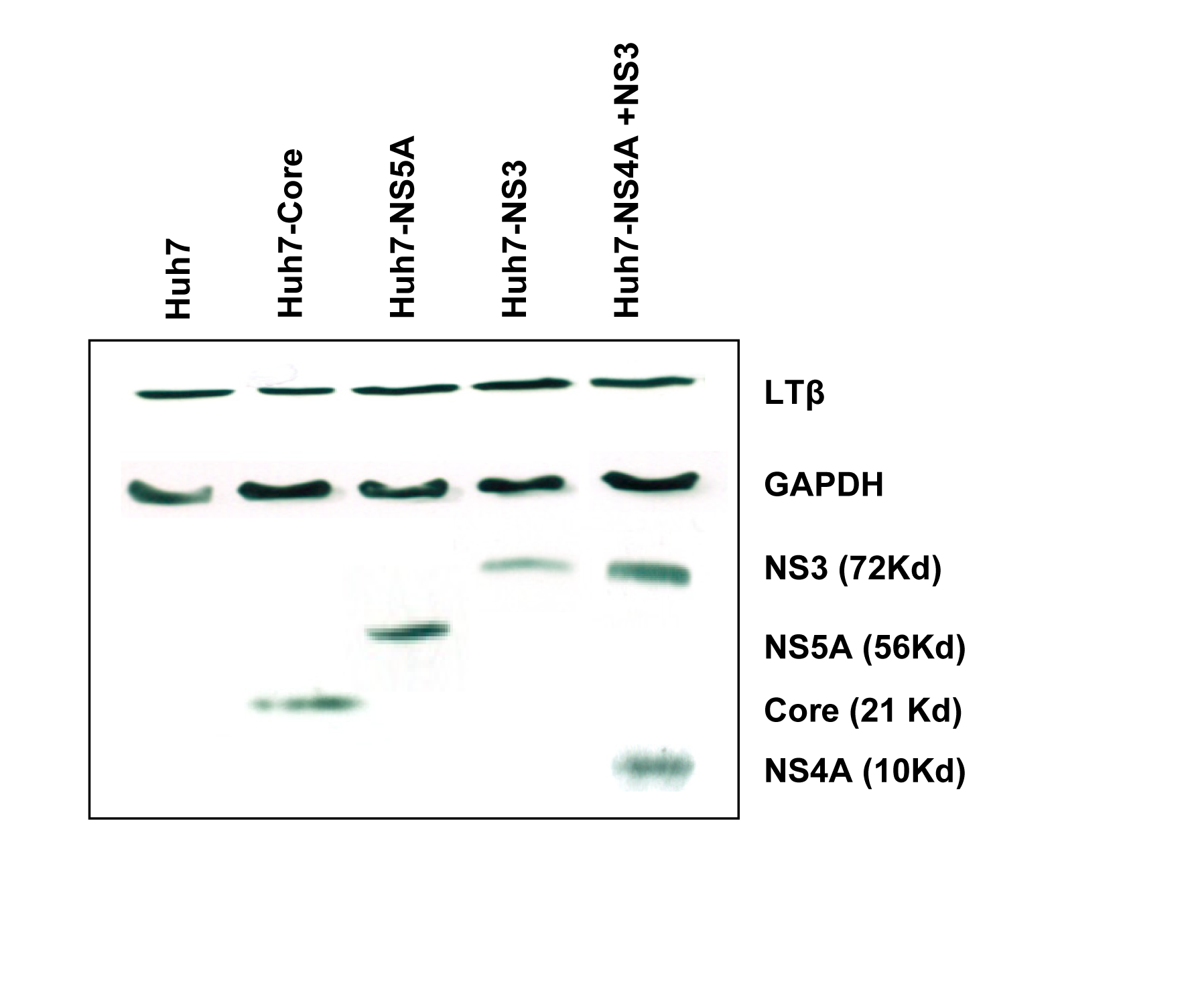

Supplement: Figure S6 — LTβ expression in cell lines stably expressing individual HCV proteins. Huh7 cells were transduced with retroviral vectors coding for myc-tagged HCV1b proteins NS3, NS4A, core and NS5A, as indicated. Viral proteins expression was revealed by immunoblotting with an anti-myc monoclonal antibody. (TIF) [file ppat.1003234.s006.tif]

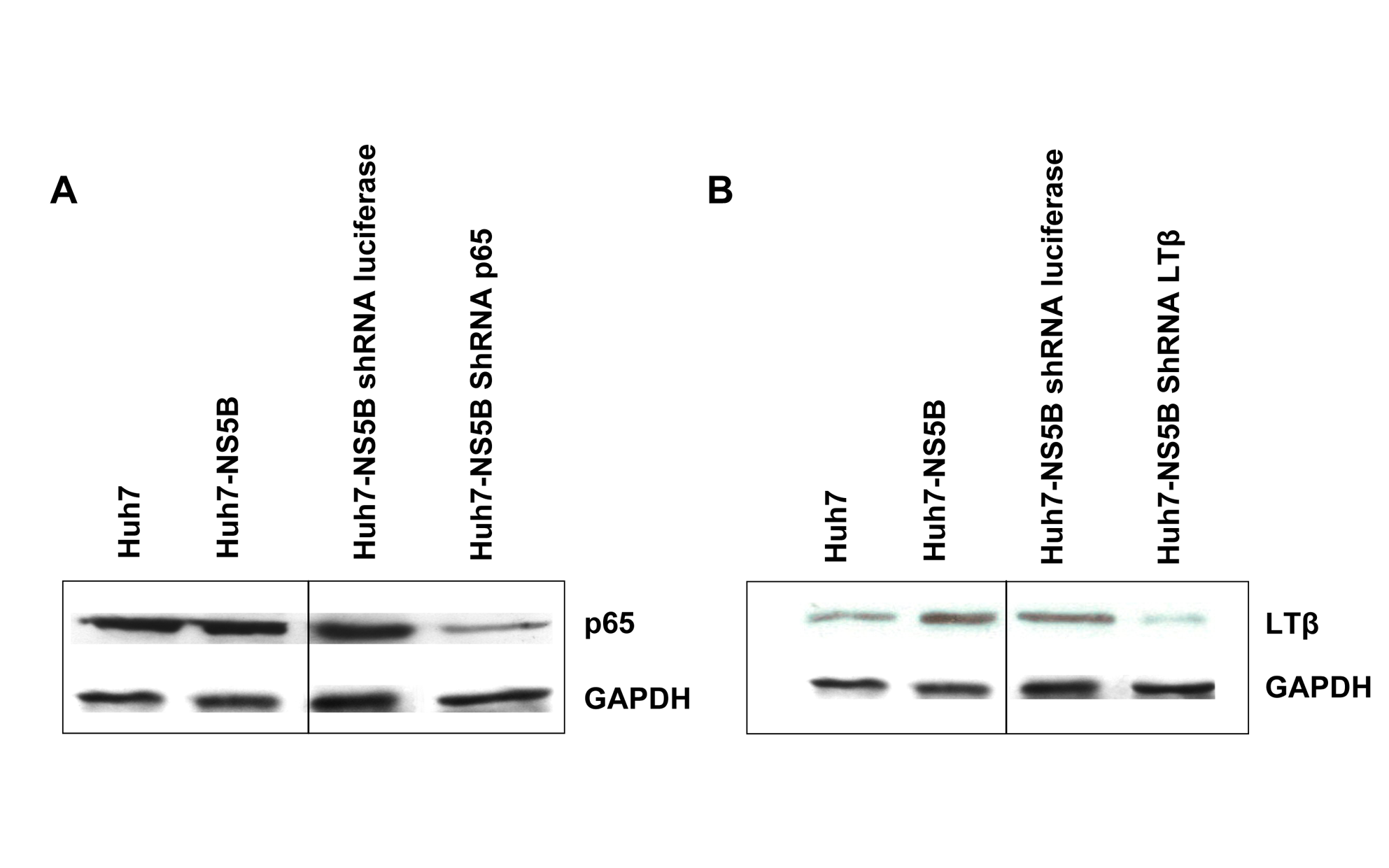

Supplement: Figure S7 — p65 and LTβ are efficiently silenced by their cognate shRNA. NS5B-expressing and parental Huh7 cells were transduced with retroviral vectors encoding shRNA for p65 (A) or LTβ (B) and protein expression was assayed by immunoblotting. GAPDH served as a loading control. (TIF) [file ppat.1003234.s007.tif]
